# Lignin Extracted from Various Parts of Castor (*Ricinus communis* L.) Plant: Structural Characterization and Catalytic Depolymerization

**DOI:** 10.3390/polym15122732

**Published:** 2023-06-19

**Authors:** Yihan Wang, Shihao Su, Guoyong Song

**Affiliations:** 1 Beijing Key Laboratory of Lignocellulosic Chemistry, Beijing Forestry University, Beijing 100083, China; y.wang7@outlook.com (Y.W.); sushihao1994@126.com (S.S.); 2 Engineering Research Center of Forestry Biomass Materials and Energy, Ministry of Education, Beijing Forestry University, Beijing 100083, China

**Keywords:** castor plant, lignin, structural characterization, catalytic depolymerization

## Abstract

Castor is an important non-edible oilseed crop used in the production of high-quality bio-oil. In this process, the leftover tissues rich in cellulose, hemicellulose and lignin are regarded as by-products and remain underutilized. Lignin is a crucial recalcitrance component, and its composition and structure strongly limit the high-value utilization of raw materials, but there is a lack of detailed studies relating to castor lignin chemistry. In this study, lignins were isolated from various parts of the castor plant, namely, stalk, root, leaf, petiole, seed endocarp and epicarp, using the dilute HCl/dioxane method, and the structural features of the as-obtained six lignins were investigated. The analyses indicated that endocarp lignin contained catechyl (C), guaiacyl (G) and syringyl (S) units, with a predominance of C unit [C/(G+S) = 6.9:1], in which the coexisted C-lignin and G/S-lignin could be disassembled completely. The isolated dioxane lignin (DL) from endocarp had a high abundance of benzodioxane linkages (85%) and a low level of β-β linkages (15%). The other lignins were enriched in G and S units with moderate amounts of β-O-4 and β-β linkages, being significantly different from endocarp lignin. Moreover, only *p*-coumarate (*p*CA) incorporated into the epicarp lignin was observed, with higher relative content, being rarely reported in previous studies. The catalytic depolymerization of isolated DL generated 1.4–35.6 wt% of aromatic monomers, among which DL from endocarp and epicarp have high yields and excellent selectivity. This work highlights the differences in lignins from various parts of the castor plant, providing a solid theory for the high-value utilization of the whole castor plant.

## 1. Introduction

Castor (*Ricinus communis* L.) is a fast-growing, robust perennial shrub belonging to the Euphorbiaceae family and is mainly distributed throughout tropical and semi-tropical areas [1,2,3,4]. This plant shows high adaptability to different climates, making it one of the most important bioenergy crops in the world. Currently, the major castor-producing states are India, Mozambique, Brazil and China. It is estimated that the global planting area of castor was about 1.3 million hectares in 2018, with the production of around 2.8 million tons [2]. The castor plants consist mainly of seeds, stalks, leaves and roots, of which the seeds account for ca. 46.8% by weight. It is reported that there is about 46–55 wt% oil in castor seeds, which is usually regarded as raw materials for the production of high-quality bio-oil, being extensively used in industrial fields [3,5]. Therefore, the castor plant is considered to be an important non-edible oilseed crop for biodiesel production. During the production of castor oil, the leftover tissues, including castor stalk, root, leaf, seed endocarp and epicarp, are underutilized as by-products, resulting in the waste of resources and environmental pollution [6]. Hence, improving the utilization rate of castor plants would be conducive to improving the economy of the castor industry.

Castor plants, as lignocellulosic biomass, are primarily composed of cellulose, hemicellulose and lignin, in which carbohydrates (cellulose and hemicellulose), as cell wall polysaccharides, can be used as sustainable feedstock for the production of low-cost chemicals, biofuels, and materials [7,8,9,10,11,12]. However, the existence of the physical entanglements between the three components, especially the lignin–carbohydrate complex linkages, impedes the efficient conversion of polysaccharides for valorization [13,14,15]. As a result, the utilization of carbohydrates for valorization is highly dependent on the lignin content, composition and structure. Generally, lignin is a complex aromatic heteropolymer synthesized mainly from *p*-coumaryl alcohol, coniferyl alcohol and sinapyl alcohol [16,17,18,19,20,21]. The oxidative coupling of three monolignols produces *p*-hydroxyphenyl (H), guaiacyl (G) and syringyl (S) units, respectively, linked by C-O and C-C bonds, resulting in β-O-4, β-β, and β-5 substructures within the polymer. The content, composition and structure of natural lignin vary widely depending on the species of plants or the tissues of the same plants [16]. For example, softwood lignin consists mainly of G and S units, hardwood lignin is composed principally of G units, while grass lignin contains G, S and H units. In addition to common lignins, a novel and naturally occurring catechyl lignin (C-lignin) was recently discovered in the seed endocarps of some plants, such as Vanilla, Cactaceae and Euphorbiaceae plants [19,22,23,24,25,26,27]. It was found that C-lignin is a homogeneous polymer produced by oxidative polymerization of simplex caffeyl alcohol, with benzodioxane being the main linkage. Compared with common lignins, C-lignin has the advantages of linearity and acid resistance, making it an “ideal lignin” archetype for valorization [28,29]. The above scenarios indicated that knowledge of the physical composition and chemical structure of lignin in biomass is a prerequisite for designing an effective pretreatment strategy for delignification.

Despite the fact that more attention has been paid to the production of castor oil, there is a lack of detailed studies on the composition and structure of the lignin extracted from castor plants. As reported in previous studies, researchers have focused on the endocarp lignin of castor seeds, which is composed of G/S-lignin and C-lignin [23,27,29], but there are fewer reports describing the lignin chemistry in other parts of castor plants. Recently, we reported that dilute HCl in 1,4-dioxane could act as an effective pretreatment method for the disassembling of C-lignin and G/S-lignin from *Jatropha* seeds endocarp [25]. Considering the high separation efficiency, the protocol was used in this study to extract lignin samples from various tissues of castor plants, including the stalk, root, leaf, petiole, seed endocarp and epicarp. Subsequently, compositional identification and structural characterization of the as-obtained lignins were performed via a series of analytical techniques, including thioacidolysis, nuclear magnetic resonance (NMR) (^31^P and 2D HSQC) and gel permeation chromatography (GPC) analyses. Furthermore, the results of catalytic depolymerization of the different lignin samples were evaluated.

## 2. Materials and Methods

### 2.1. Materials

The castor plants were obtained from Shandong Province, China. Pd/C (Pd content: 5 wt%) and 1,4-dioxane were purchased from Energy Chemical, Shanghai, China. All of the commercially available chemical reagents were used without further purification. In this work, the stalk, root, leaf, petiole, seed endocarp and epicarp of castor plants were separated manually. The as-obtained materials were ground into powders (20–40 mesh) and were then dewaxed with ethanol/toluene (*v*/*v* = 1:2) for 10 h. The collected samples were dried and stored in the desiccator for further analysis.

### 2.2. Lignin Isolation

The 1,4-dioxane lignin (DL) samples of castor plants were obtained according to our previous study [25]. Typically: taking castor stalk as an example, the dewaxed sample (10 g) was extracted with 1,4-dioxane (100 mL, [HCl] = 18 mmol/L) at 85 °C for 3 h. The soluble fraction was acquired via filtration and then concentrated to provide a thick solution, which was added dropwise into pH = 2.0 solution to form a precipitate. As a result, the castor stalk DL was obtained by centrifugation, washed with deionized water until neutral, and freeze-drying.

### 2.3. Structural Characterization

Thioacidolysis analysis. The reaction of the lignin sample (30 mg) with BF_3_-etherate (0.2 M, 5 mL) in a dioxane/ethanethiol (*v*/*v* = 8.75:1) was carried out at 100 °C for 4 h [23,30]. Upon completion, the lignin oily products were first obtained via the removal of volatiles treated with *N*, *O*-bis(trimethylsilyl)trifluoroacetamide (BSTFA) in anhydrous THF at 65 °C for 1 h under N_2_, and then subjected to GC/GC-MS analyses (GC-2010 or GC/MS-QP2010SE, Shimadu, Japan).

GPC analysis. The lignin sample was treated with acetic anhydride/pyridine (*v*/*v*=1:1, 1.0 mL) in a bottle at room temperature for 72 h. The acetylated sample was obtained by extraction and evaporation, was then dissolved in THF (*ca.* 2 mg/mL) and filtered with a PTFE filter (0.45 μm) before GPC analysis. The average molecular weight of the acetylated lignin was determined using LC-20AD (PL-gel 10 μm Mixed-B 7.5 mm I.D. column and UV detection detector) (Shimadzu, Kyoto, Japan).

NMR analysis. For 2D HSQC NMR analysis, the lignin sample (50 mg) was dissolved in DMSO-*d*_6_ (0.5 mL) and analyzed using a Bruker Avance spectrometer (Rheinstetten, Germany) among which the solvent peak of DMSO-*d*_6_ at δ_C_/δ_H_ = 39.5/2.49 ppm was used as an internal reference. The detailed parameters were obtained from the previous report [31]. The spectral widths were 5000 Hz and 20,000 Hz for the ^1^H- and ^13^C-dimensions, respectively. The number of collected complex points was 1024 for ^1^H-dimension with a recycle delay of 1.5 s. The number of transients was 64, and 256 time increments were always recorded in the ^13^C-dimension. The ^1^*J*_CH_ used was 145 Hz. Prior to Fourier transformation, the data matrixes were zero filled up to 1024 points in the ^13^C-dimension. Data processing was performed using standard Bruker Topspin-NMR 4.1.4 software. For ^31^P NMR analysis, a lignin sample (20 mg) was dissolved in anhydrous pyridine and deuterated chloroform (700 μL, *v*/*v* = 1.6:1) with cyclohexanol (1.09 mg) as an internal standard and chromium (III) acetylacetonate (2 mg) as a relaxation reagent. The mixture was then treated with 2-chloro-4,4,5,5-tetramethyl-1,3,2-dioxaphospholate (TMDP, 100 μL) for about 10 min and was transferred into a 5 mm NMR tube for analysis (Bruker Avance spectrometer, 400 MHz).

GC/GC-MS analyses. The identification and quantification of aromatic monomers in lignin oily products derived from thioacidolysis and catalytic hydrogenolysis were performed on Shimadu GC-2010 and GC/MS-QP2010SE, respectively [32]. Specifically, GC-MS analysis of lignin oily products was performed on Shimadu GC/MS-QP2010SE equipped with a HP-5 MS capillary column and a mass spectroscopy detector. Helium was used as a carrier gas, with a constant column flow of 1 mL/min. The injector temperature was held constant at 250 °C. The injector, interface and ion source temperature were set at 250 °C, 280 °C and 200 °C, respectively. The split ratio was 50:1, and the scan model was applied to identify the aromatic monomers from 50 *m*/*z* to 700 *m*/*z*. The oven temperature was programmed from 50 °C to 280 °C with 8 °C/min and held at 50 °C and 280 °C for 3 min and 5 min, respectively. GC analysis of lignin oily products was conducted on Shimadu GC-2010 equipped with an HP-5 column and a flame ionization detector (FID). Nitrogen was used as a carrier gas with a constant column flow of 1.39 mL/min. The injector temperature and the detection temperature (FID) were held at 250 °C and 290 °C, respectively. The split ratio was 20:1, and the temperature programming was consistent with GC-MS. The identification and quantification of lignin monomers in the oily products were assessed via a comparison with authentic samples.

### 2.4. Catalytic Hydrogenolysis of Lignin into Aromatics

In a typical catalytic reaction, the hydrogenolysis of lignin (50 mg) with Pd/C (10 mg) in methanol (10 mL) was carried out in a 50 mL Parr autoclave. The reactor was purged with N_2_, inflated with H_2_ (3 MPa) at room temperature, and was then heated to 240 °C for 4 h under magnetic stirring (600 rpm). After the completion of the reaction, the autoclave was cooled and depressurized carefully. The lignin oily product was obtained via filtration and the removal of all of the volatiles and was then subjected to GC/GC-MS analyses. Particularly, the as-obtained lignin oily products from castor seed endocarp should be silylated with excess BSTFA in anhydrous THF at 65 °C for 1 h under N_2_ before GC/GC-MS analyses.

## 3. Results and Discussion

### 3.1. Chemical Compositional Analysis of Various Castor Parts

Initially, the castor stalk, root, leaf, petiole, seed endocarp and epicarp fractions were separated manually and analyzed independently. NREL biomass compositional analysis was used to determine the contents of the main constituents in each sample [33]. The detailed results are shown in Table 1 and Table S1. The results indicated there are significant differences in lignin content between the six samples, among which the root and stalk had higher lignin contents (25.7% and 21.0%, respectively) than the other three fractions (epicarp, leaf and petiole). It is worth noting that the NREL method is not suitable for the determination of the lignin content in seed coat because the existence of fatty acids and proteins in the seeds would lead to the overestimation of the true lignin level [19,34]. Therefore, the lignin content of castor seed endocarp is shown for reference only. Except for seed endocarp, all castor fractions had a high content of carbohydrates (cellulose and hemicellulose), ranging from 54 to 70 wt%, which could be used as a sustainable substrate for the production of bioethanol. For the further determination of lignin composition in the whole-cell walls of all samples, thioacidolysis analysis was carried out (Figure S1). Aromatic monomers released from thioacidolysis were identified as α, β, γ-trithioethylpropyl-substituted derivatives, which could be determined via GC-MS. The results indicated that castor seed endocarp contained C-lignin and G/S-lignin, with a high ratio of C-monomer and G/S-monomers [C/(G+S) = 6.9:1], being slightly higher than that from previous reports [25]. Compared with seed endocarp, only G/S-lignin was detected in the remaining castor tissues. However, the ratio values of G/S from lignin in these samples were significantly different, ranging from 0.4 to 2.7 (Figure S1). The above scenarios revealed that there is an obvious difference in the composition and structure of lignins in various parts of castor plants.

### 3.2. Lignin Isolation

**Figure 1:**
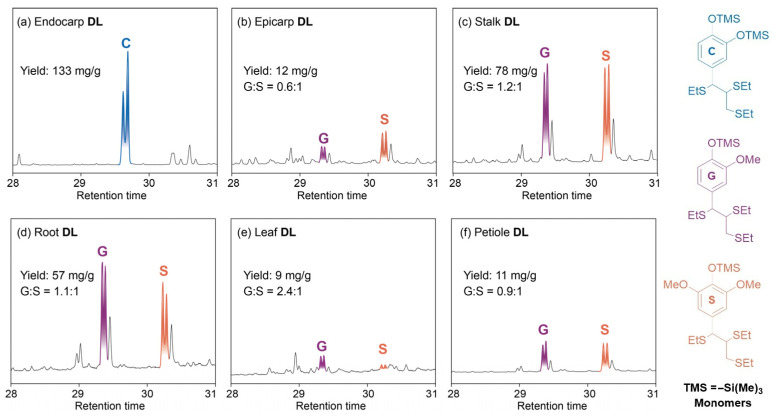
GC-MS spectra of thioacidolysis products from different DL samples.

In this study, the dilute HCl in the 1,4-dioxane method was used to isolate lignin samples from various parts of castor plants, namely endocarp DL, epicarp DL, stalk DL, root DL, leaf DL and petiole DL. Compared with the other five lignin samples, the DL isolated from castor seed endocarp had higher yield (133 mg/g) and lower carbohydrate level (6.3 wt%), being consistent with our previous results (Figure 1 and Table S2) [25]. However, the yields of DL isolated from castor seed epicarp, leaf and petiole were relatively lower (12 mg/g, 9 mg/g and 11 mg/g, respectively), which might be due to the low level of lignin in the parent materials. Subsequently, the detailed structural characterization of extracted lignin samples was further analyzed using a combination of chemical methods and spectral techniques.

### 3.3. Thioacidolysis Analysis

The DL samples from various tissues of castor plants were analyzed through the use of thioacidolysis, and the results are shown in Figure 1. Concerning the treatment of endocarp DL with BF_3_-etherate in a dioxane/ethanethiol, the GC-MS spectra showed that there was only an obvious doublet signal ascribed to catechyl (C)-derived monomeric products while no signals for G/S-lignin were observed. This scenario revealed that the C-lignin and G/S-lignin coexisting in castor seed endocarp were disassembled completely, being in line with a previous report [25]. However, when epicarp DL, stalk DL, root DL, leaf DL and petiole DL were used as substrates, and guaiacyl (G) and syringyl (S)-derived monomers were determined in all lignin samples, indicating that the G/S-lignin in castor plants could also be separated by using this isolation protocol. The analysis above showed that C-lignin could be preferentially isolated from castor seed endocarp under current conditions, which might be attributed to the low molecular weight and high level of C-lignin in seed endocarp [22,26].

### 3.4. 2D HSQC NMR Analysis

**Figure 2:**
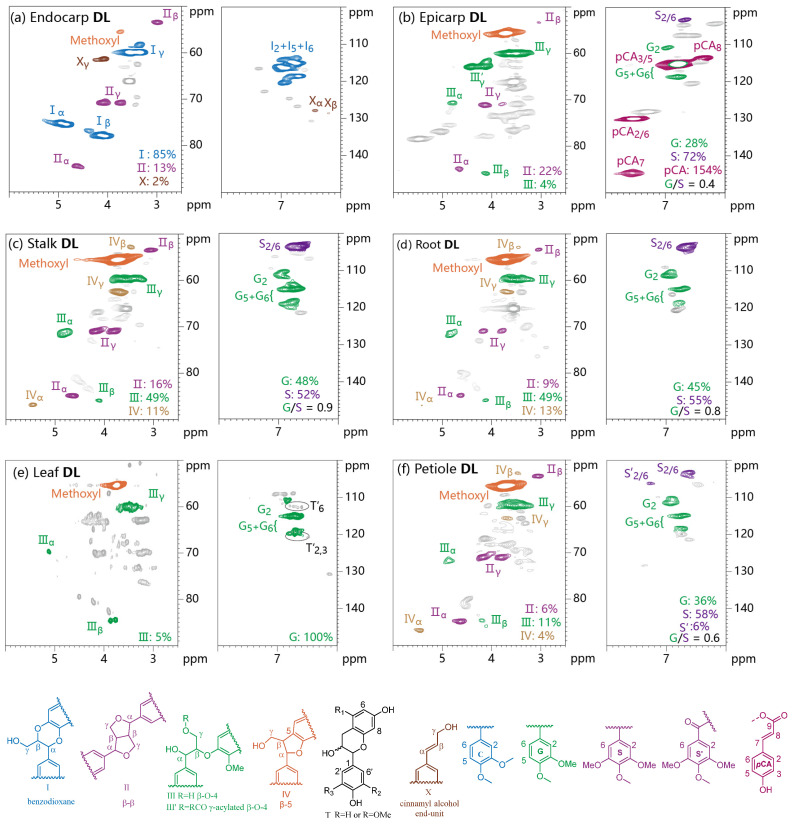
Expanded aliphatic side chain and aromatic regions of 2D NMR spectra of different DL samples.

Two-dimensional HSQC NMR technology is widely considered a rapid and efficient strategy used to obtain useful structural information on lignin macromolecular structure [35,36,37]. The 2D HSQC spectra of various lignin samples and the main substructures of lignin are shown in Figure 2 and Figure S2, Table S3. In the side chain region (δ_C_/δ_H_ 50–90/2.5–6.0 ppm) of NMR spectra, the cross-signals of internal linkages in lignin were highlighted. As expected, the presence of benzodioxane linkages (I, 85%, labelled in blue) is pronounced in the spectrum of endocarp DL. The less abundance of resinol linkages (II, 13%, labelled in purple) and cinnamyl alcohol end-units (X, 2%, labelled in brown) were also detected, with no observation of the signals for β-O-4 linkages. Among them, the cross signals of α, β and γ in benzodioxane linkages were located at δ_C_/δ_H_ 75.6/4.93, 78.2/4.13 and 60.1/3.29–3.76 ppm, respectively. The above cases for castor endocarp DL are consistent with the results from the thioacidolysis analysis, and are also in accordance with the published literature [25]. In the case of other five DL samples, the signals of β-O-4 linkages (III, III’ labelled in green) resonated at δ_C_/δ_H_ 71.4/4.89, 86.4/4.15, 60.8/3.21–3.95 and 63.2/4.31 ppm were omnipresent [37,38], but their relative abundance is significantly different, among which the highest value of β-O-4 linkages per 100 aromatic units was found in stalk DL and root DL (49%). The analysis indicated that the contents of β-O-4 linkages in lignins derived from various parts of castor plants strongly varied and were highly dependent on the lignification degree of plant tissues. As for resinol linkages, the cross signals were detected in epicarp DL (22%), stalk DL (16%), root DL (9%) and petiole DL (6%) but absent in leaf DL. In addition, phenylcoumaran linkage (IV, labelled in yellow) was observed only in some DL samples, such as stalk DL, root DL and petiole DL.

In the aromatic region (δ_C_/δ_H_ 100–150/6.0–8.0 ppm) of HSQC spectra of the six DL samples, the peaks of catechyl (C), guaiacyl (G) and syringyl (S) units could be detected for identification of lignin composition (Figure 2). In the spectrum of endocarp DL, the prominent cross-signals were ascribed to C-units, which are located at 115.9/6.80, 117.6/6.94 and 119.8/6.90 ppm, being in line with the results of thioacidolysis analysis. Less abundant but clear signals of unsaturated linkages in cinnamyl alcohol end-units were also observed. Compared with endocarp DL, G and S components were detected in other lignin samples, except for leaf DL, with them only having G units. The lack of observation of the signals of S units in the NMR spectrum of leaf DL might be due to its lower abundance. It should be noted that the trance of the signals derived from tannins were also observed in the spectrum of leaf DL according to the reported literature [39]. The ratio values of G/S calculated by NMR were close to those from thioacidolysis analysis of the parent materials but slightly lower than the values from corresponding DL samples. In addition to the G and S units, the cross-signal peaks from *p*-coumarate (*p*CA) were also observed in the spectrum of epicarp DL, which is a remarkable feature that differs from other lignin samples. The high abundance of *p*CA in epicarp DL makes it a potential substrate for the production of *p*-coumaric acid. This scenario is also different from lignin in common gramineous plants, which usually contain the two hydroxycinnamic acid moieties, i.e., *p*-coumaric (*p*CA) and ferulic acid (FA) [40,41].

### 3.5. ^31^P NMR Spectra

^31^P NMR is considered a powerful tool for distinguishing and quantifying the lignin functional groups, including aliphatic alcohols, phenolic hydroxyl and carboxylic acid groups [42,43]. The quantitative results expressed in mmol of functional groups per gram of dry lignin (mmol/g) are shown in Table 2, Table S4 and Figures S3–S8. In the ^31^P NMR spectrum of leaf DL, because the signals of hydroxyl groups derived from lignin and condensed tannins might have overlapped [42], this case would not be discussed here. The amount of aliphatic OH in five lignin samples ranged from 2.38 to 3.32 mmol/g, with the highest level in petiole DL and the lowest content in endocarp DL. As for phenolic OH, a clear signal peak for catechol OH appeared at about 138.0–140.0 ppm in the spectrum of endocarp DL, and the content was calculated as 2.43 mmol/g. Compared with endocarp DL, the other five DL featured guaiacyl OH and syringyl OH, among which the ratios of both them are basically consentient with the results obtained from thioacidolysis and 2D HSQC analyses. It was worth noting that higher amounts of phenolic OH from *p*-coumarate were also observed in epicarp DL [44,45]; this scenario was also verified via 2D HSQC analysis.

### 3.6. Molecular Weights Analysis

**Figure 3:**
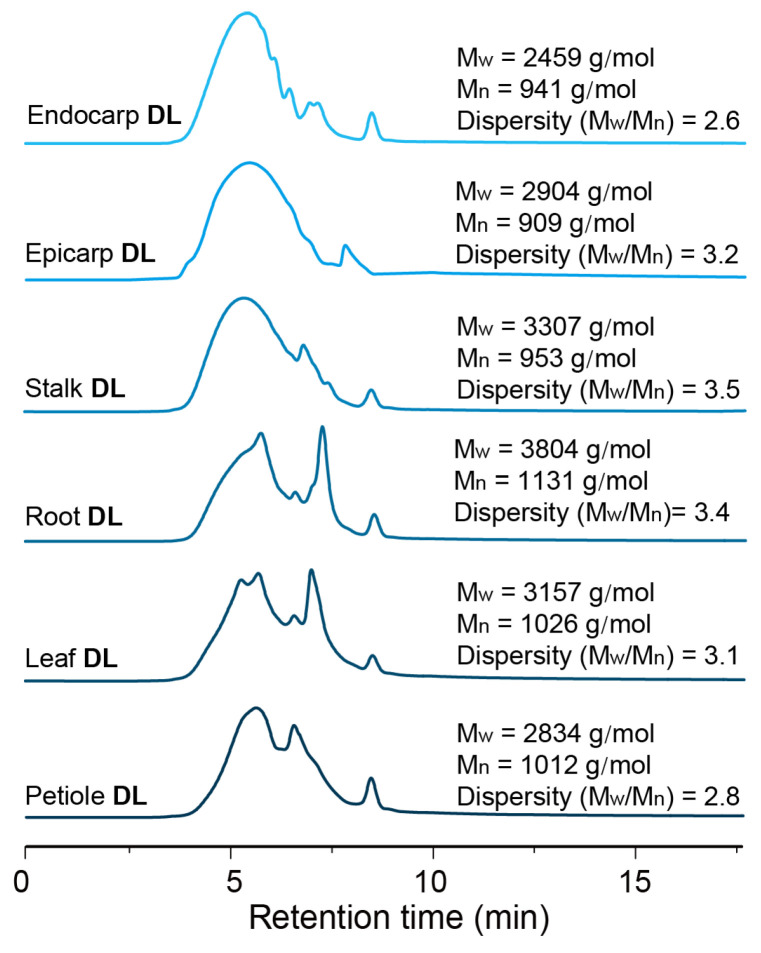
GPC spectra of different DL samples.

The molecular weights (M_w_ and M_n_) and dispersity (M_w_/M_n_) of the DL samples isolated from various parts of castor plants are shown in Figure 3. It was found that there was a slight difference in molecular weights of lignin samples, ranging from 2450 to 3810 g/mol. Among them, lignin from the endocarp of castor seeds had a lower molecular weight (endocarp DL, 2459 g/mol) when compared with the other lignin samples, being consistent with previous reports [25]. This scenario might be explained by the fact that caffeyl alcohol, an important monolignol for C-lignin synthesis, has a lower degree of polymerization than those monomers from woody and herbaceous plants, such as coniferyl alcohol, sinapyl alcohol and *p*-coumaryl alcohol [26]. In addition, all of the lignin samples had a relatively low dispersity, as shown by M_w_/M_n_ ≤ 3.5. Among them, endocarp DL had the lowest molecular weight distribution, with a dispersity of 2.6. This case indicated that it had a more homogenous structure compared to other lignin samples, being in accordance with the homogenous and linear structure of C-lignin, as reported in previous studies [19].

### 3.7. Catalytic Hydrogenolysis of Lignin into Aromatics

Having the structural information in hand, the catalytic depolymerization of various DL samples from the castor plant was performed with commercial Pd/C catalyst in methanol under 3 MPa of H_2_ at 240 °C for 4 h. Compared with standard samples, the identification and quantification of aromatic monomers in the obtained lignin oily products were carried out with GC, and the detailed results are shown in Table 3 and Figure S9. Similar to the case reported by our previous studies [27], the catalytic hydrogenolysis of endocarp DL generated 35.6 wt% of catechol monomers, in which catechylpropyl (**C1**) and catechylpropanol (**C2**) were the primary products. This selectivity of monomer towards **C2** reached 99% in all monomeric products. The molecular weight of lignin oily products from the depolymerization of endocarp DL had a significant decrease when compared with parent lignin (2459 g/mol) (Figure 3 and Figure S10). Being different from endocarp DL, the depolymerization of epicarp DL afforded low-molecular-weight lignin oily products (638 g/mol), from which small numbers of aromatic monomers (*ca.* 0.2 wt%) were from β-O-4 units and large amounts of methyl 3-(4-hydroxyphenyl) propanoate (**H1**, 15.8 wt%) were from hydroxycinnamic acid moiety. The excellent selectivity of monomer towards **H1**, in this case, could be explained by the low abundance of β-O-4 linkages and the high level of *p*-coumarate (*p*CA) component in epicarp DL, as demonstrated via 2D HSQC NMR. This scenario also indicated that castor epicarp might serve as a promising substrate for the production of *p*-coumarate and its derivates. In the cases of stalk DL and root DL, moderate monomers yields were observed (11.4 wt% and 8.4 wt%, respectively), in which all aromatics are derived from β-O-4 units, being composed of guaiacyl (G) and syringyl (S) derivates. The ratio values of G-monomers and S-monomers were slightly lower than the results from 2D HSQC NMR, revealing that the S-monomers are more easily released from lignin macromolecule, being in line with previous reports [46,47]. GPC analysis also showed an obvious decrease in their molecular weight (699 g/mol and 633 g/mol, respectively) (Figure S10). When leaf DL and petiole DL were used as substrates, only 1.4 wt% and 4.0 wt% of phenolic monomers were observed, but the molecular weights were low (597 g/mol and 530 g/mol, respectively) (Figure S10). In addition to aromatic products, the long-chain fatty acid from lipids was also detected in a higher amount (2.4–6.2 wt%). The relatively low monomer yields might be due to the low abundance of β-O-4 linkages and high content of lipids in leaf DL and petiole DL samples.

## 4. Conclusions

In summary, the first detailed structural characterization of lignins isolated from various tissues of castor plants was performed by using a combination of chemical methods and spectral techniques, describing the differences in chemical composition and structure. Thioacidolysis coupled with NMR spectroscopy suggested that endocarp DL consists mainly of a C unit with a high abundance of benzodioxane linkages (85%), and the other DL samples are composed principally of G and S units with higher contents of β-O-4 bonds (4–49%) and lower level of β-β linkages (6–22%). In addition, epicarp DL has a significant feature of high amounts of *p*-coumarate (*p*CA), which differs from other DL samples. Catalytic depolymerization of six DL samples provides 1.4–35.6 wt% of aromatic monomers, among which DL from endocarp and epicarp have high yields and excellent selectivity, being conducive to subsequent separation and purification. This work highlights the differences between lignins from various parts of castor plants, which will contribute to the efficient utilization of this resource for the production of high-value chemicals, thereby improving the economy of the castor industry.

## Figures and Tables

**Table 1:** The composition (cellulose, hemicellulose, and lignin) and lignin subunit distributions of various castor plants tissues ^a^.

Entry	Tissues	Cellulose (wt%)	Hemicellulose (wt%)	Lignin (wt%)	Lignin Subunit Distributions (mol%) ^c^		
					C	G	S
1	Endocarp	19.3	10.6	13.3 ^b^	87	5	8
2	Epicarp	42.1	27.2	16.8	−	29	71
3	Stalk	46.2	24.0	21.0	−	47	53
4	Root	44.7	19.5	25.7	−	47	53
5	Leaf	35.5	19.0	13.8	−	73	27
6	Petiole	37.7	17.7	11.1	−	47	53

^a^ The compositions of biomass were analyzed according to the procedures of the NREL method. ^b^ Based on the dilute HCl in 1,4-dioxane method. ^c^ Based on the thioacidolysis analysis–Signifies not detected.

**Table 2:** The hydroxyl groups contents of various DL samples (millimoles per gram).

DL Samples	Aliphatic OH	Guaiacyl OH		Syringyl OH		*p*CA-OH	Catechol OH	Carboxylic Group	Total Phenolic OH
		Cond. ^a^	Non. Cond. ^a^	Cond. ^a^	Non. Cond. ^a^				
Endocarp	2.38	− ^b^	−	−	−	−	2.43	0.02	2.43
Epicarp	2.70	0.03	0.16	0.08	0.02	1.32	−	0.02	1.61
Stalk	2.72	0.08	1.00	0.16	0.69	−	−	0.01	1.93
Root	2.64	0.29	1.22	0.10	0.37	−	−	0.02	1.98
Petiole	3.32	0.19	0.53	0.45	0.53	−	−	0.02	1.70

^a^ “Cond. and Non. Cond.” represent condensed phenolic units and noncondensed phenolic units, respectively. ^b^ Signifies not detected.

**Table 3:** Catalytic depolymerization of various DL samples from castor plants over Pd/C catalyst.

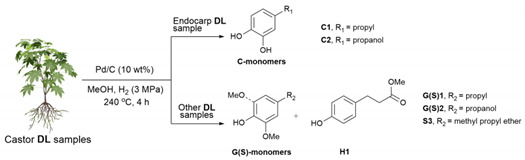											
DL Samples	Phenolic Monomers (wt%) ^a^								Total Yields (wt%) ^a^	G/S ^a^	M_w_ (g/mol)
	C1	C2	G1	G2	S1	S2	S3	H1			
Endocarp	3.2	32.4	− ^b^	−	−	−	−	−	35.6	−	510
Epicarp	−	−	0.2	trace	trace	trace	trace	15.8	16.0	−	638
Stalk	−	−	1.2	2.0	1.6	1.8	4.8	−	11.4	0.5	699
Root	−	−	1.0	1.8	1.0	1.4	3.2	−	8.4	0.6	633
Leaf	−	−	0.8	0.6	−	−	−	−	1.4	−	597
Petiole	−	−	0.4	1.0	0.8	0.2	2.0	−	4.4	1.0	530

Reaction conditions: Samples (50 mg), Pd/C (10 mg), MeOH (10 mL), H_2_ (3 MPa), 240 °C, 4 h. ^a^ Determined by comparison with authentic samples on GC. ^b^ Signifies not detected.

## Data Availability

Not applicable.

## Supplementary Materials

The following supporting information can be downloaded at: https://www.mdpi.com/article/10.3390/polym15122732/s1, Figure S1: GC spectra of thioacidolysis products from different parts of castor; Figure S2: 2D NMR spectra of different DL samples; Figure S3–S8: Quantitative ^31^P NMR spectra of various DL sample; Figure S9: Products distribution of Pd/C-catalyzed depolymerization of various castor DL samples; Figure S10: GPC spectra of lignin oily products from Pd/C-catalyzed hydrogenolysis of various DL samples of castor plants. Table S1: The contents of acid-insoluble lignin (AIL), acid-soluble lignin (ASL), total lignin content (TLC), cellulose, hemicellulose and extraction of the various raw materials; Table S2: The contents of acid insoluble lignin (AIL), acid soluble lignin (ASL), total lignin content (TLC), cellulose, and hemicellulose of the various DL samples; Table S3: NMR data for the signal assignments for DL samples from various parts of castor in DMSO-*d*_6_; Table S4: Typical chemical shifts and integration regions for DL samples from various parts of castor in the ^31^P NMR spectrum.
