# Supplementary material for: Lignin Extracted from Various Parts of Castor (Ricinus communis L.) Plant: Structural Characterization and Catalytic Depolymerization
Source: Polymers (Basel). 2023 Jun 19;15(12):2732. doi: 10.3390/polym15122732 (PMC10300757; doi:10.3390/polym15122732)
Supplement: Supplementary file 1 [file polymers-15-02732-s001.zip › polymers-2431653-supplementary.pdf]

polymers

MDPI

Supplementary Materials

Lignin Extracted from Various Parts of Castor (*Ricinus communis L.*) Plant: Structural Characterization and Catalytic Depolymerization

Yihan Wang [1,†], Shihao Su [1,†] and Guoyong Song [1,2,*]

[1] Beijing Key Laboratory of Lignocellulosic Chemistry, Beijing Forestry University, Beijing 100083, China; y.wang7@outlook.com (Y.W.); sushihao1994@126.com (S.S.)
[2] Engineering Research Center of Forestry Biomass Materials and Energy, Ministry of Education, Beijing Forestry University, Beijing 100083, China
* Correspondence: songg@bjfu.edu.cn
† These authors contributed equally to this work.

Table S1. The contents of acid insoluble lignin (AIL), acid soluble lignin (ASL), total lignin content (TLC), cellulose, hemicellulose and extraction of the various raw materials.[a.]

Samples	AIL	ASL	TLC	Cellulose	Hemicellulose	Extraction
Endocarp	59.5	1.1	13.3[b]	19.3	10.6	9.1
Ericarp	14.4	2.4	16.8	42.1	27.2	2.5
Stalk	19.0	2.0	21.0	46.2	24.0	3.1
Root	21.8	3.9	25.7	44.7	19.5	3.8
Leaf	12.2	1.6	13.8	35.5	19.0	6.8
Petiole	8.9	2.2	11.1	37.7	17.7	4.5

[a] The compositions of biomass were analyzed according to the procedures of the NREL method. [b] Based on the dilute HCl in 1,4-dioxane method.

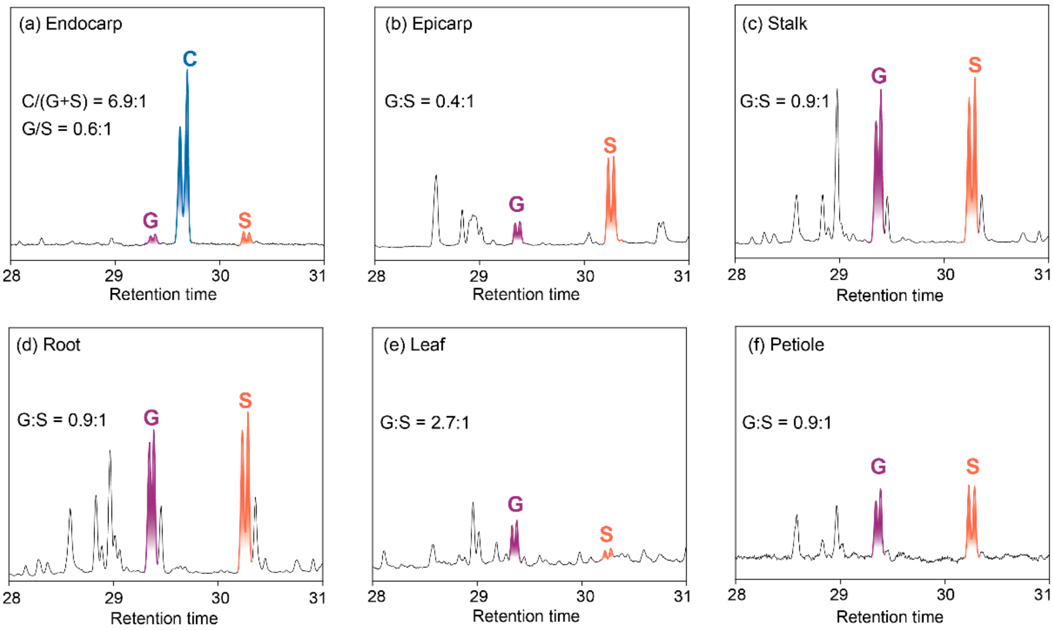

Figure S1. GC spectra of thioacidolysis products from different parts of castor.

Table S2. The contents of acid insoluble lignin (AIL), acid soluble lignin (ASL), total lignin content (TLC), cellulose, and hemicellulose of the various DL samples.

Samples	AIL	ASL	TLC	Cellulose	Hemicellulose	Total
Endocarp	85.0	1.6	86.6	3.4	2.9	92.9
Epicarp	62.8	6.2	69.0	3.1	2.3	74.4
Stalk	75.3	1.4	76.7	8.2	1.6	86.5
Root	70.3	3.3	73.6	7.1	0.5	81.2
Leaf	52.0	6.4	58.4	5.9	3.5	67.8
Petiole	57.2	6.0	63.2	6.2	2.0	71.4

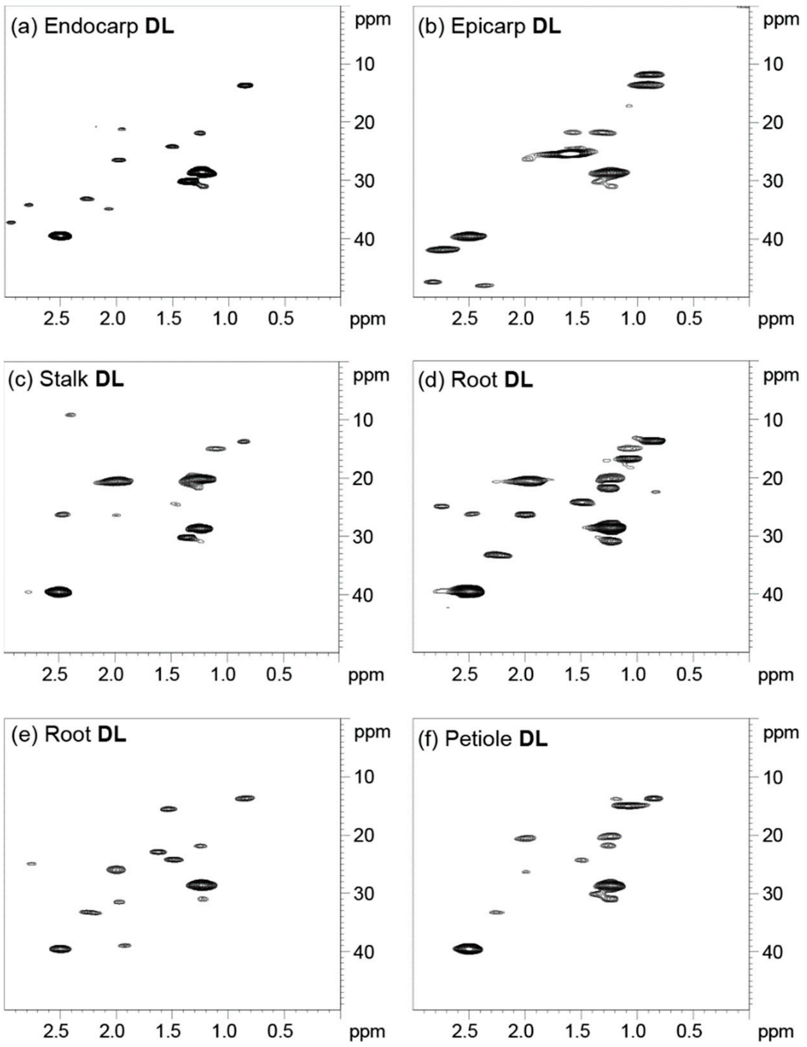

Figure S2. 2D NMR spectra of different DL samples.

Table S3. NMR data for the signal assignments for DL samples from various parts of castor in DMSO-d_6.

Lable	δ_C/δ_H (ppm)	Assignment
I_α	75.6/4.93	C_α-H_α in benzodioxane substructures (I)
I_β	78.2/4.13	C_β-H_β in benzodioxane substructures (I)
I_γ	60.1/3.29-3.76	C_γ-H_γ in benzodioxane substructures (I)
II_α	84.5/4.62	C_α-H_α in β-β resinol substructures (II)
II_β	53.4/3.04	C_β-H_β in β-β resinol substructures (II)
II_γ	70.7/3.74-4.09	C_γ-H_γ in β-β resinol substructures (II)
III_α	71.4/4.89	C_α-H_α in β-O-4 substructures (III)
III_β	86.4/4.15	C_β-H_β in β-O-4 substructures (III)
III_γ	60.8/3.21-3.95	C_γ-H_γ in β-O-4 substructures (III)
IV_α	86.5/5.48	C_α-H_α in phenylcoumaran (IV)
IV_β	53.2/3.47	C_β-H_β in phenylcoumaran (IV)
IV_γ	62.3/3.88	C_γ-H_γ in phenylcoumaran (IV)
X_α	127.9/6.42	C_α-H_α in cinnamyl alcohol end-units (X)
X_β	128.7/6.20	C_β-H_β in cinnamyl alcohol end-units (X)
X_γ	61.4/4.10	C_γ-H_γ in cinnamyl alcohol end-units (X)
G_2	110.7/6.92	C_2-H_2 in guaiacyl units (G)
G_5	114.5/6.68	C_5-H_5 in guaiacyl units (G)
G_6	119.0/6.77	C_6-H_6 in guaiacyl units (G)
$S_{2/6}$	103.5/6.68	$C_{2/6}$-$H_{2/6}$ in syringyl units (S)
$S'_{2/6}$	106.2/7.3	$C_{2/6}$-$H_{2/6}$ in oxidized syringyl units (S`)
pCA$_{3/5}$	115.5/6.76	$C_{3/5}$-$H_{3/5}$ in *p*-coumarate (*p*CA)
pCA$_{2/6}$	130.5/7.45	$C_{2/6}$-$H_{2/6}$ in *p*-coumarate (*p*CA)
pCA$_7$	144.6/7.47	C_7-H_7 in *p*-coumarate (*p*CA)
pCA$_8$	113.9/6.22	C_8-H_8 in *p*-coumarate (*p*CA)
I_2, I_5, I_6	115.9/6.80, 117.6/6.94, 119.8/6.90	C_2-H_2, C_5-H_5, C_6-H_6, in catechol units (I)

Table S4. Typical chemical shifts and integration regions for DL samples from various parts of castor in the ^{31}P NMR spectrum.

Structure	δ (ppm)
Aliphatic OH	145.4–150
Condensed Guaiacyl OH	141.42–142.17
Noncondensed Guaiacyl OH	138.79–140.17
Condensed Syringyl OH	143.2–144.5
Noncondensed Syringyl OH	142.17–143.2
p-coumarate (*p*CA) OH	136.7–137.8
Catechol OH	138.0–140.0
Carboxylic group	134.2–135.5

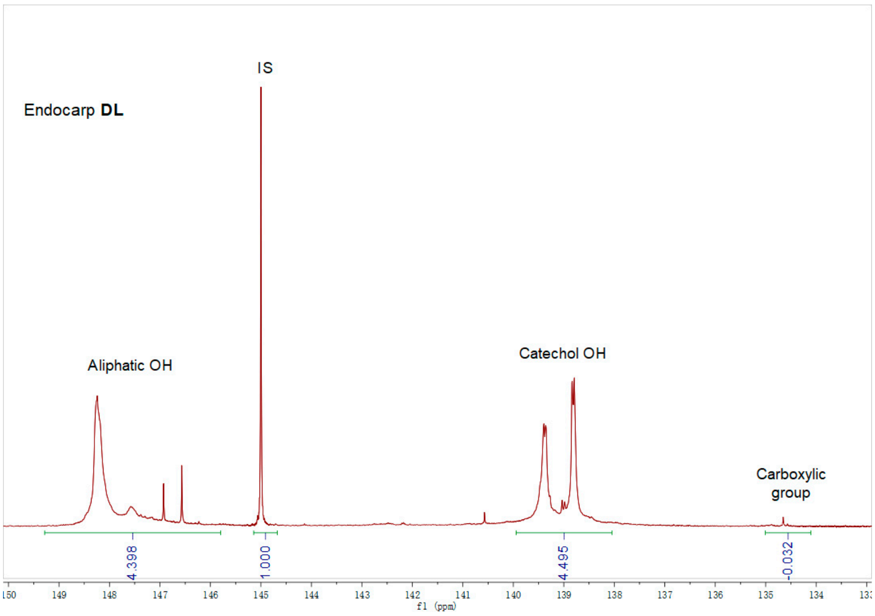

Figure S3. Quantitative ^{31}P NMR spectrum of the Endocarp DL sample.

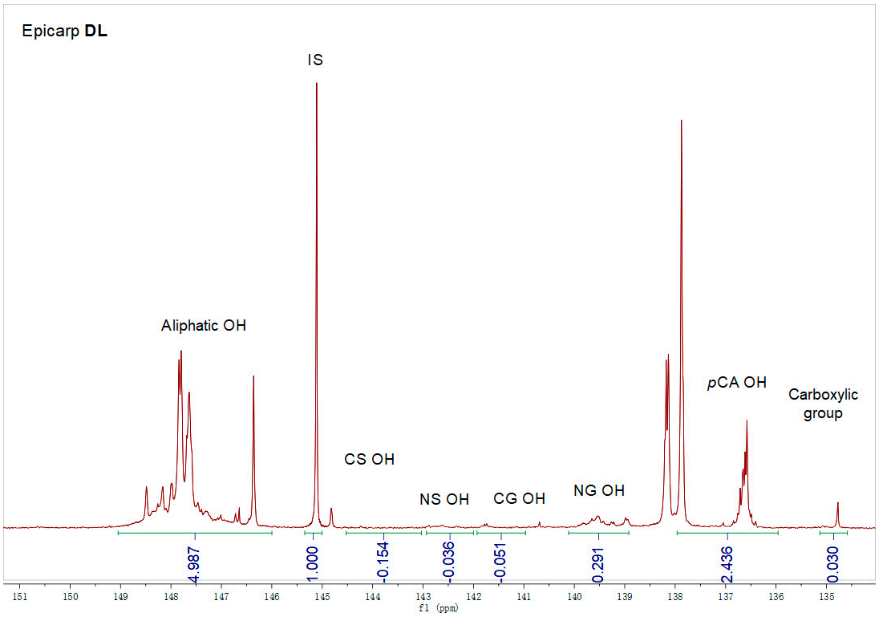

Figure S4. Quantitative ^{31}P NMR spectrum of the Epicarp DL sample.

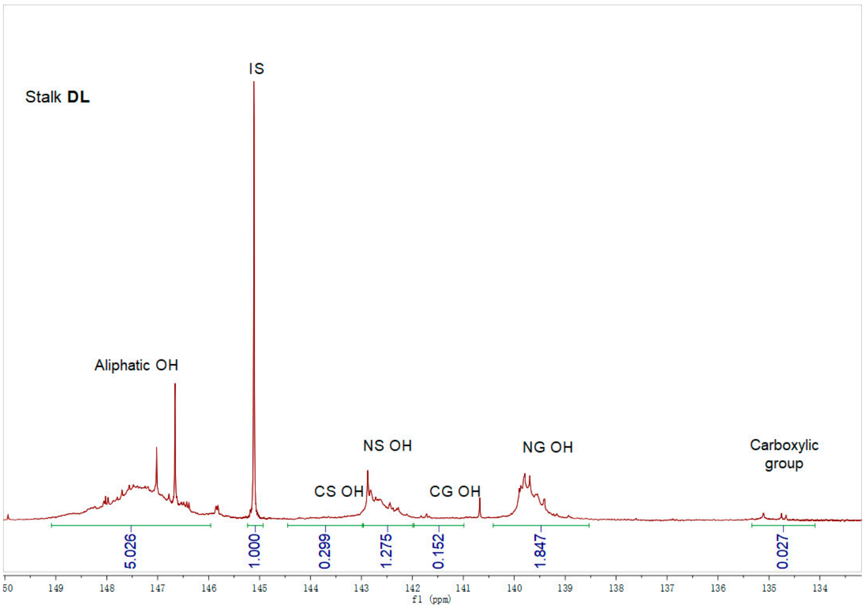

Figure S5. Quantitative ^{31}P NMR spectrum of the Stalk DL sample.

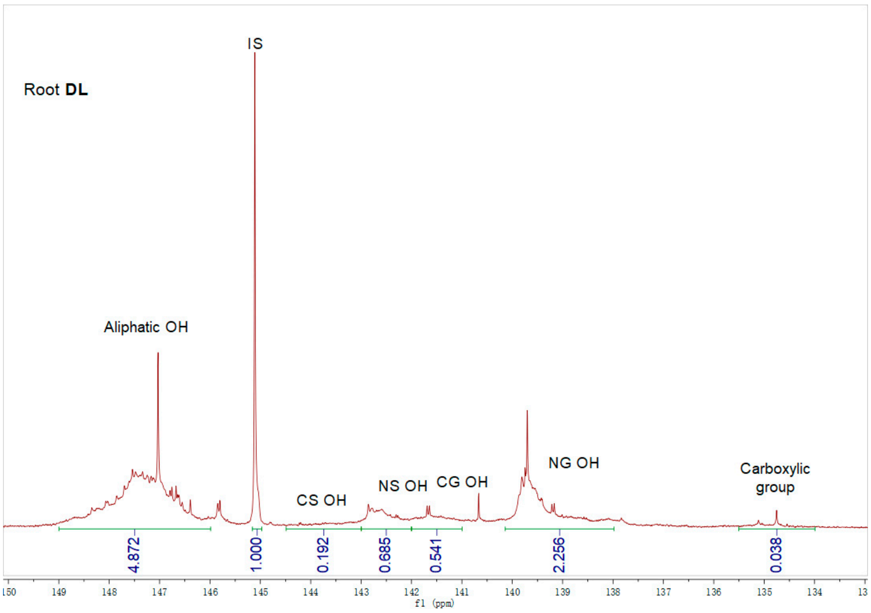

Figure S6. Quantitative ^{31}P NMR spectrum of the Root DL sample.

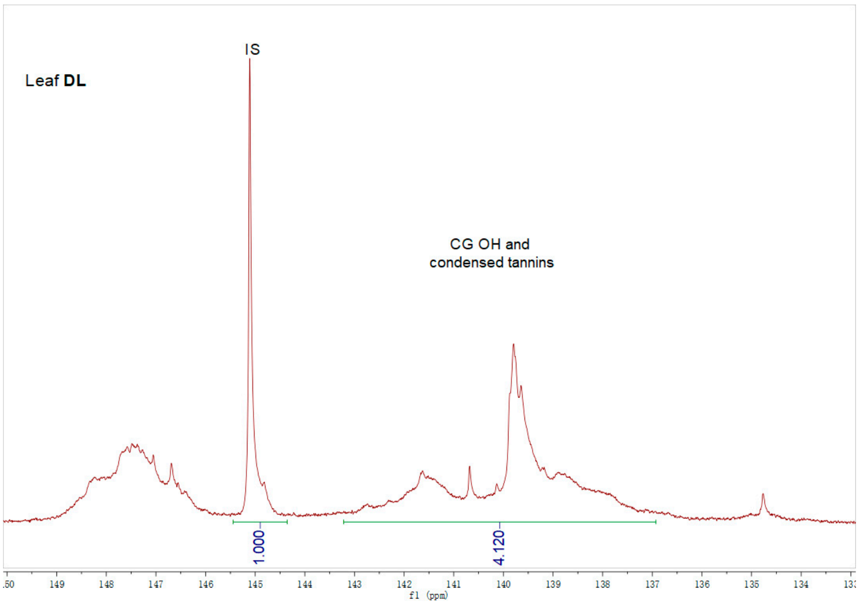

Figure S7. Quantitative ^{31}P NMR spectrum of the Leaf DL sample.

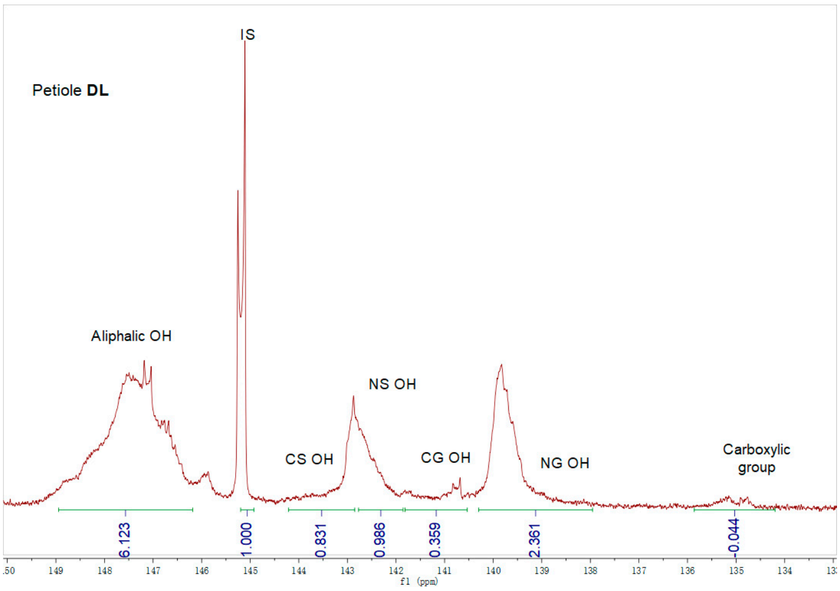

Figure S8. Quantitative ^{31}P NMR spectrum of the Petiole DL sample.

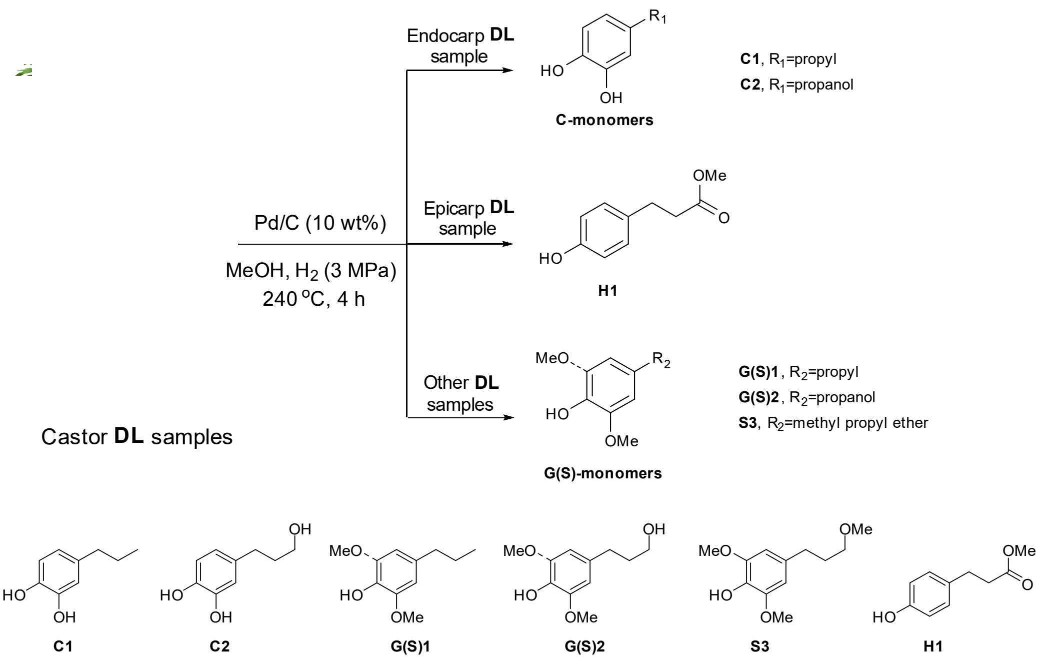

Figure S9. Products distribution of Pd/C-catalyzed depolymerization of various castor DL samples.

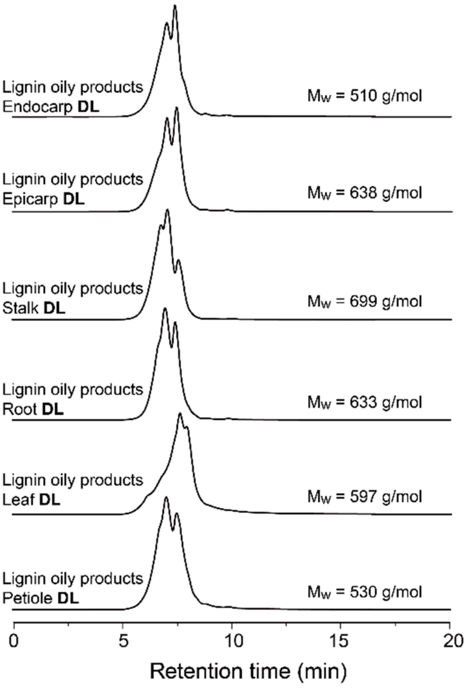

Figure S10. GPC spectra of lignin oily products from Pd/C-catalyzed hydrogenolysis of various DL samples of castor plants. Reaction conditions: sample (50 mg), Pd/C (10 mg), MeOH (10 mL), H_2 (3 MPa), 240 °C, 4 h.
